# Effectiveness and safety of tafluprost in primary open-angle glaucoma and ocular hypertension: a post-marketing phase IV study in China

**DOI:** 10.1186/s12886-022-02553-1

**Published:** 2022-08-05

**Authors:** Xinghuai Sun, Qinghuai Liu, Xin Tang, Ke Yao, Yan Li, Jin Yang, Mingchang Zhang, Huiping Yuan, Yan Zheng, Weining Li, Huacong Peng

**Affiliations:** 1 Department of Ophthalmology and Visual Science, Eye and ENT Hospital, Shanghai Medical College, Fudan University, Shanghai, 200031 China; 2 Department of Ophthalmology, The First Affiliated Hospital of Nanjing Medical University, Nanjing, 210029 Jiangsu Province China; 3 Beijing Ophthalmology and Visual Sciences Key Laboratory, Beijing Tongren Eye Center, Beijing Tongren Hospital, Capital Medical University, Beijing, 100730 China; 4 Eye Center, Second Affiliated Hospital, Zhejiang University School of Medicine, Hangzhou, 310009 Zhejiang Province China; 5 Department of Ophthalmology, First Affiliated Hospital of Kunming Medical University, Kunming, 650031 Yunnan Province China; 6 Tianjin Eye Institute, Tianjin Key Lab of Ophthalmology and Visual Science, Tianjin Eye Hospital, Nankai University Affiliated Eye Hospital, Tianjin, 300020 China; 7 Department of Ophthalmology, Tongji Medical College, Union Hospital, Huazhong University of Science & Technology, Wuhan, 430030 Hubei Province China; 8 Department of Ophthalmology, The Second Affiliated Hospital, Harbin Medical University, Harbin, 150001 Heilongjiang Province China; 9 Department of Ophthalmology, Xinhua Hospital Affiliated to Shanghai Jiao Tong University School of Medicine, Shanghai, 200092 China; 10 Department of Ophthalmology, Qilu Hospital, Cheeloo College of Medicine, Shandong University, Jinan, 250012 Shandong Province China; 11 Department of Cataract and Glaucoma, Wuhan Eyegood Ophthalmic Hospital, Wuhan, 430064 Hubei Province China

**Keywords:** Tafluprost, Primary open-angle glaucoma, Ocular hypertension, Clinical trial, Phase IV, China

## Abstract

**Background:**

Prostaglandin analogs (PGAs) are the first-line treatment for primary open-angle glaucoma (POAG) and ocular hypertension (OH). This study aimed to confirm the effectiveness and safety of Tapros® (0.0015% tafluprost eye drops) in Chinese patients with POAG and OH.

**Methods:**

This phase IV, multicenter, non-comparative, prospective study enrolled patients with POAG and OH in China between 12/27/2017 and 04/15/2020. Patients who were treatment-naïve or untreated within one month (group A) or with unreached intraocular pressure (IOP) target after previous monotherapy of other PGAs (group B) or non-PGA IOP-lowering drugs (group C) were treated with 0.0015% tafluprost for three months. The IOP reduction, response rate, and safety were observed.

**Results:**

There were 165, 89, and 31 patients in groups A, B, and C, with baseline IOPs of 22.4 ± 4.7, 21.0 ± 3.5, and 22.5 ± 3.2 mmHg, respectively. The least-square means and percentages of IOP reduction at 3 months for groups A, B, and C were 4.7 (19.8%), 1.6 (6.1%), and 4.6 mmHg (20.3%), respectively. A significant reduction in IOP was observed at each visit compared with baseline (all *P* < 0.05). At the final visit, 57.0% of the participants in group A achieved an IOP reduction of ≥ 20%, while 40.4% and 77.4% in groups B and C achieved an IOP reduction of ≥ 10%. Fifty-eight treatment-related adverse events occurred in 46 participants (15.7%), of which the most common one was conjunctival hyperemia (34/293, 11.6%).

**Conclusions:**

Tafluprost showed a sustained and significant effect with tolerable adverse events in Chinese patients with POAG and OH who were treatment-naïve or untreated within one month or received prior treatments with unsatisfying outcomes.

## Background

Glaucoma is a major cause of irreversible blindness worldwide [1]. Age is an important risk factor for glaucoma [2]. The proportion of older adults in China is rising, with the share of the population aged ≥ 65 years increased from 8.9% in 2010 to 13.5% in 2020 [3], which is lower than the caucasian population (16.89% in the United States, 18.65% in the United Kingdom, 20.69% in the European Union, etc.) [4]. In China, glaucoma was affecting 21 million individuals in 2020, among whom 5.67 million would be blind [5]; the prevalence of all glaucoma, primary open-angle glaucoma (POAG), and primary angle-closure glaucoma (PACG) is 2.6%, 1.0%, and 1.4% [6]. Elevated intraocular pressure (IOP) is the most important factor for the progression of glaucoma and the only one that can be modified. Lowering IOP is beneficial for glaucoma and ocular hypertension (OH), and medication for ophthalmic use is the first choice [7, 8].

Prostaglandin analogs (PGAs) are recommended as first-line IOP-lowering drugs for POAG and OH as they provide remarkable effects, along with little systemic side effects and convenience in administration [9–14]. Tafluprost is a novel PGA with a high and selective binding affinity for the prostanoid FP receptor [15]. Several randomized controlled trials (RCTs) verified the efficacy of tafluprost on lowering IOP in patients with POAG and OH, alongside its favorable safety profile [16, 17]. Moreover, a significant IOP reduction at each time point compared with baseline during circadian monitoring after receiving tafluprost was observed not only in patients with POAG and OH [18] but also normal-tension glaucoma (NTG) [19]. Consistent with the RCTs, observational studies conducted worldwide also confirmed the effectiveness and safety of tafluprost for POAG and OH in real-world settings [13, 20–24].

Following a phase III RCT [25], tafluprost was approved for marketing and available in 2015 in China. The mean IOP of healthy Chinese was 15.0 ± 2.8 mmHg [26], lower than that of a healthy population in other races [27, 28]. Furthermore, 70% of the Chinese patients with POAG have NTG and exhibit an upper limit of IOP lower than 21 mmHg [29]. It contrasts with Western populations, in which patients with POAG exhibit high IOP [7]. Nevertheless, the relatively high baseline IOP in Ge’s study [25] did not represent the circumstances in actual clinical practice where POAG is mostly featured by normal or relatively low IOP. Arguably, with the paucity of relevant studies with a large sample size, little is known about the generalized effect of tafluprost in the Chinese population in real-world practice.

Therefore, this study aimed to confirm the effectiveness and safety of tafluprost for Chinese patients with POAG and OH.

## Methods

### Study design and participants

This prospective, multicenter, non-comparative, phase IV study enrolled patients with POAG and OH who were treatment-naïve or untreated within 1 month or with unreached target IOP after previous IOP-lowering monotherapy between December 27, 2017 and April 15, 2020 from 11 centers in China. The study was approved by the Ethics Committee of Eye and ENT Hospital, Shanghai Medical College, Fudan University (Approval No. 2017008–1).; the Ethics Committee of Beijing Tongren Hospital, Capital Medical University (Approval No. TREC2017-62); the Ethics Committee of The Second Affiliated Hospital, Harbin Medical University (Approval No. 2017-MD-023); the Ethics Committee of Tianjin Eye Hospital (Approval No. TJYYLL-2017–11); the Ethics Committee of Xinhua Hospital Affiliated to Shanghai Jiao Tong University School of Medicine (Approval No. XHEC-C-2017–037-2); the Ethics Committee of First Affiliated Hospital of Kunming Medical University (Approval No. 2017-YL-29); the Ethics Committee of The First Affiliated Hospital of Nanjing Medical University (Approval No. 2017-MD-214); the Ethics Committee of Qilu Hospital, Shandong University (Approval No. 2017040); the Ethics Committee of Tongji Medical College, Union Hospital, Huazhong University of Science & Technology (Approval No. 2017–232); the Ethics Committee of The Second Affiliated Hospital, Zhejiang University School of Medicine (Approval No. 2017–430); the Ethics Committee of Wuhan Eyegood Ophthalmic Hospital (Approval No. AG-BNZ-201805). All methods were performed in accordance with the relevant guidelines and regulations. Informed consent was obtained from all subjects involved in the study.

The inclusion criteria were 1) Chinese patients of ≥ 18 years of age, 2) confirmed with POAG or OH who had not received any IOP-lowering drugs within at least a month or with unreached target IOP (IOP still ≥ 17 mmHg) when using a single IOP-lowering drug, and 3) willing to participate and signed the informed consent. The exclusion criteria were 1) uncontrolled systemic comorbidities (e.g., hypertension, diabetes, and chronic kidney disease) or active non-glaucomatous eye diseases, 2) known hypersensitivity or contraindications to any component of the study drug, 3) corneal lesions precluding accurate tonometry reading, 4) participation in other trials within 30 days, 5) pregnancy, lactation or preparing for pregnancy, 6) IOP ≥ 35 mmHg in either eye for treatment-naïve patients and patients untreated within a month, IOP > 28 mmHg or < 17 mmHg for pre-treated patients, 7) received mixture or combined anti-glaucoma therapy in the past month, 8) history of intraocular surgery within three months, or 9) unsuitable for participating in the study according to the ophthalmologist’s judgment. If both eyes of a participant met the eligibility criteria, the one with higher IOP was the target eye. If both eyes had equal IOP, the right eye was selected.

The study was approved by the ethics committee of each center. All participants provided written informed consent. This study was conducted in conformance with the Good Clinic Practice and adhered to the Declaration of Helsinki. The study was registered in chictr.org.cn (No. ChiCTR-OIC-17011980).

### Treatment

The participants were grouped according to their previous treatment: 1) group A, treatment-naïve or untreated within 1 month, 2) group B, receiving another PGA monotherapy with IOP still ≥ 17 mmHg, and 3) group C, receiving IOP-lowering monotherapy other than PGAs with IOP still ≥ 17 mmHg.

All participants were given one drop of 0.0015% tafluprost (Tapros®, containing benzalkonium chloride 0.1 mg/ml) once daily in the evening for 3 months. Participants in groups B and C discontinued their previous drugs and directly switched to tafluprost without a washout period.

### Data collection

Demographic characteristics, medical history, ophthalmic history, and medication history were recorded before initiating tafluprost. Visual field examinations, anterior chamber gonioscope, and optical coherence tomography (OCT) were performed at enrollment as optional based on the ophthalmologist’s judgment. Visual acuity, slit-lamp examination, and IOP were performed at enrollment and all visits. OCT was performed as optional after 3 months of treatment.

IOP was measured twice (or three times if the measurements differed by > 3 mmHg) at each time-point by the Goldmann applanation tonometer, and the average value was used for analysis. Whenever a third time was measured, the average value was calculated by the two times with the closest value.

### Follow-up and outcomes

Follow-up data, including ophthalmic examination, visual field examinations, and IOP, were collected at 1 week (7 ± 2 days), 1 month (4 ± 1 weeks), and 3 months (12 ± 2 weeks) after starting tafluprost.

The outcomes included the average degree and percentage of IOP reduction at 1 week, 1 month, and 3 months of tafluprost treatment compared with baseline. The response rate was defined as the proportion of participants achieving an IOP reduction of ≥ 20% and ≥ 30% in group A and an IOP reduction of ≥ 10% in groups B and C. Patients with baseline IOP ≤ 21 mmHg and ≥ 24 mmHg in group A were analyzed. Patients achieving an IOP of ≤ 16, ≤ 18, and ≤ 20 mmHg at the final visit in group A were analyzed. In groups B and C, the proportion of participants achieving an IOP reduction of ≥ 1 mmHg was assessed.

The safety profile included ocular and systemic adverse events (AEs). AEs were recorded throughout the study.

### Statistical analysis

Using PASS 15.0.5, a sample size of 148 yields a two-sided 95% confidence interval of IOP reduction at 3 months of treatment from baseline with a distance from the mean to the limits of 0.65 mmHg when the estimated standard deviation was 4 mmHg. Considering a dropout rate of 10%, a sample size of 165 was required. Furthermore, in order to obtain more information on the safety profile of tafluprost, we included not only treatment-naïve patients but also those treated with IOP-lowering drugs other than tafluprost, and expanded the sample size to 300 in order to observe AEs with incidence > 1%.

The full analysis set (FAS) was used to analyze all outcomes. The FAS included the participants who received tafluprost at least once and had at least one follow-up IOP measurement. Missing data were filled by the last observation carries forward method. The safety set (SS) included all participants who received tafluprost at least once and had any safety evaluation data.

The statistical analysis was performed using SAS 9.4 (SAS Institute, Cary, NY, USA). Continuous variables are presented as means ± standard deviations for data with a normal distribution and were analyzed using the paired t-test, and otherwise presented as medians (ranges) and analyzed using Wilcoxon’s rank test. Categorical variables are presented as numbers (percentages) and were compared using the chi-square test or Fisher’s exact test. An analysis of covariance was used to calculate the least squares mean (Lsmeans) and 95% confidence intervals of IOP reduction at follow-up from baseline, with baseline IOP as the covariant. All statistical tests were two-sided. *P* < 0.05 were considered statistically significant.

## Results

### Study population and patient characteristics

Finally, 300 participants were enrolled. Among them, 293 participants were included in the SS (group A, *n* = 170; group B, *n* = 91; group C, *n* = 32), and 285 participants were included in the FAS (group A, *n* = 165; group B, *n* = 89; group C, *n* = 31). Among patients in SS, eight participants withdrew since none of the follow-ups was involved. All patients were evaluated after 1 week and 1 month of treatment. At 3 months after treatment, nine participants in group A and 1 in group C were lost to follow-up due to the COVID-19 pandemic. Four participants in group A and three in group B withdrew because they thought the treatment efficacy was unsatisfactory. Nine, eight and one patient in groups A, B and C withdrew upon investigator’s judgment or due to AEs.

Among the participants in the FAS, the mean age was 44.5 ± 16.1 (range, 18–89) years, and 188 participants (66.0%) were male. Most (88.8%, 253/285) participants used IOP-lowering drugs in both eyes. About half of the patients (49.8%, 142/285) had comorbidities. The baseline IOPs were 22.4 ± 4.7, 21.0 ± 3.5, and 22.5 ± 3.2 mmHg in groups A, B, and C, respectively. In group A, 47.9% and 38.2% of the participants had baseline IOP of ≤ 21 and ≥ 24 mmHg, respectively (Table 1). The most common comorbidities were hypertension (A/B/C: *n* = 23/9/6), ametropia (A/B/C: *n* = 28/3/2), dry eye (A/B/C: *n* = 15/5/4), diabetes (A/B/C: *n* = 10/9/1), cataract (A/B/C: *n* = 7/6/3), and autoimmune rheumatic disease (A/B/C: *n* = 5/4/2).

**Table 1:** Demographic data of the participants

Item	All (*N* = 285)	Group A (*N* = 165)	Group B (*N* = 89)	Group C (*N* = 31)	P
Age (y)	44.5 ± 16.1	43.7 ± 16.0	45.3 ± 17.1	46.5 ± 13.3	0.456
Sex					0.099
Male	188 (66.0%)	117 (70.9%)	54 (60.7%)	17 (54.8%)	
Female	97 (34.0%)	48 (29.1%)	35 (39.3%)	14 (45.2%)	
Ethnicity					0.248
Han Chinese	277 (97.2%)	160 (97.0%)	88 (98.9%)	29 (93.5%)	
Others	8 (2.8%)	5 (3.0%)	1 (1.1%)	2 (6.5%)	
With concomitant disease	142 (49.8%)	82 (49.7%)	47 (52.8%)	13 (41.9%)	0.580
Have concomitant medication	127 (44.6%)	7 (4.2%)	89 (100.0%)	31 (100.0%)	< 0.001
Current diagnosis					0.076
POAG	227 (79.6%)	136 (82.4%)	71 (79.8%)	20 (64.5%)	
OH	58 (20.4%)	29 (17.6%)	18 (20.2%)	11 (35.5%)	
Course of disease (days)					< 0.001
N (Nmiss)	232 (53)	154 (11)	58 (31)	20 (11)	
Mean ± SD	255.7 ± 786.6	101.6 ± 634.3	502.1 ± 733.8	728.1 ± 1433.7	
Eye using the study drug					0.221
Right only	21 (7.4%)	8 (4.8%)	10 (11.2%)	3 (9.7%)	
Left only	11 (3.9%)	7 (4.2%)	2 (2.2%)	2 (6.5%)	
Both	253 (88.8%)	150 (90.9%)	77 (86.5%)	26 (83.9%)	
Target eye					
Right	182 (63.9%)	101 (61.2%)	61 (68.5%)	20 (64.5%)	
Left	103 (36.1%)	64 (38.8%)	28 (31.5%)	11 (35.5%)	
Baseline IOP (mmHg)	22.0 ± 4.2	22.4 ± 4.7	21.0 ± 3.5	22.5 ± 3.2	

*POAG* Primary open-angle glaucoma, *OH* Ocular hypertension, *IOP* Intraocular pressure

### IOP reduction and response rate

**Fig. 1:**
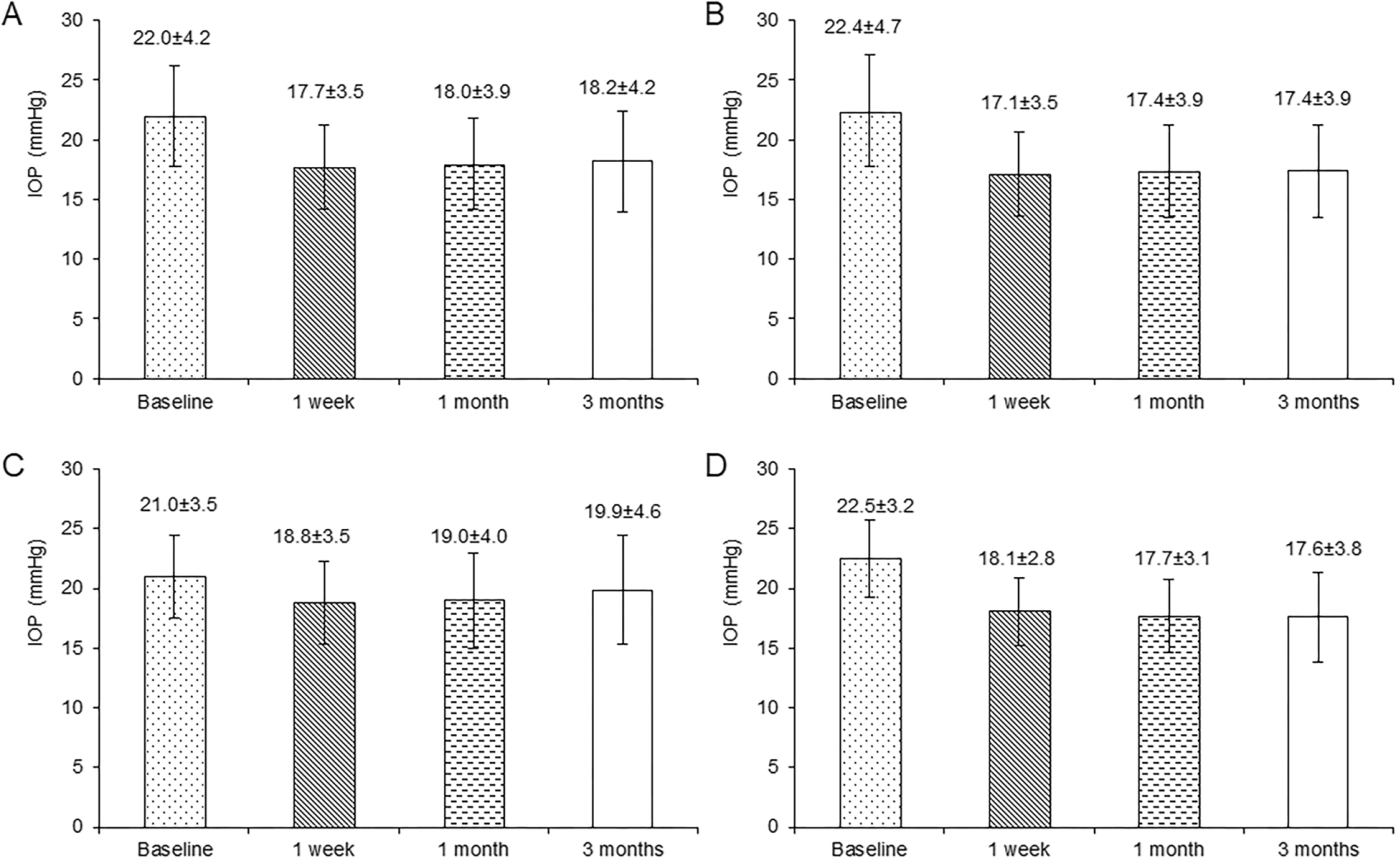
Intraocular pressure (IOP) at different time points (baseline, 1 week, 1 month, and 3 months) **A** All patients; **B** Group A; **C** Group B; **D** Group C

Of the 285 participants in the FAS, a significant reduction in IOP was achieved at 1 week, 1 month, and 3 months from a baseline level of 22.0 ± 4.2 to 17.7 ± 3.5, 18.0 ± 3.9, and 18.2 ± 4.2 mmHg, respectively (Fig. 1). IOP decreased by 4.2 (3.7–4.7), 4.0 (3.5–4.5), and 3.7 (3.2–4.3) mmHg at 1 week, 1 month, and 3 months of treatment, respectively. The IOP at each visit was significantly lower than baseline (all *P* < 0.001, Table 2).

**Table 2:** Changes in mean IOP after 1 week, 1 month, and 3 months of treatment

Time point	IOP parameter	All (*n* = 285)	Group A (*n* = 165)	Group B (*n* = 89)	Group C (*n* = 31)
Baseline	Mean IOP, mmHg	22.0 ± 4.2	22.4 ± 4.7	21.0 ± 3.5	22.5 ± 3.2
1 week	Mean IOP, mmHg	17.7 ± 3.52	17.1 ± 3.54	18.8 ± 3.47	18.1 ± 2.84
	IOP change, mmHg	4.2 (3.7–4.7)	5.0 (4.5–5.5)	2.8 (2.2–3.5)	4.1 (3.0–5.2)
	Percentage change, %	17.6 (15.8–19.5)	21.1 (19.1–23.1)	11.2 (8.4–14.0)	17.6 (12.9–22.3)
	*P* value (vs. baseline)	< 0.001	< 0.001	< 0.001	< 0.001
1 month	Mean IOP, mmHg	18.0 ± 3.9	17.4 ± 3.9	19.0 ± 4.0	17.7 ± 3.1
	IOP change, mmHg	4.0 (3.5–4.5)	4.7 (4.2–5.2)	2.5 (1.8–3.2)	4.5 (3.3–5.7)
	Percentage change, %	16.7 (14.7–18.7)	19.7 (17.4–22.0)	10.2 (7.1–13.3)	19.6 (14.4–24.9)
	*P* value (vs. baseline)	< 0.001	< 0.001	< 0.001	< 0.001
3 months	Mean IOP, mmHg	18.2 ± 4.2	17.4 ± 3.9	19.9 ± 4.6	17.6 ± 3.8
	IOP change, mmHg	3.7 (3.2–4.3)	4.7 (4.1–5.2)	1.6 (0.9–2.4)	4.6 (3.4–5.9)
	Percentage change, %	15.6 (13.5–17.7)	19.8 (17.4–22.2)	6.1 (2.8–9.4)	20.3 (14.7–25.9)
	*P* value (vs. baseline)	< 0.001	< 0.001	0.006	< 0.001

*IOP* Intraocular pressure

The IOP reduction and percentage calculated in “all patients” are arithmetic average. The IOP reduction and percentage in Group A, B, C are the least square mean (Lsmean), using the analysis of covariance model (ANCOVA), taking the baseline IOP as the covariant and the Group (A, B, C) as the fixed effect

**Fig. 2:**
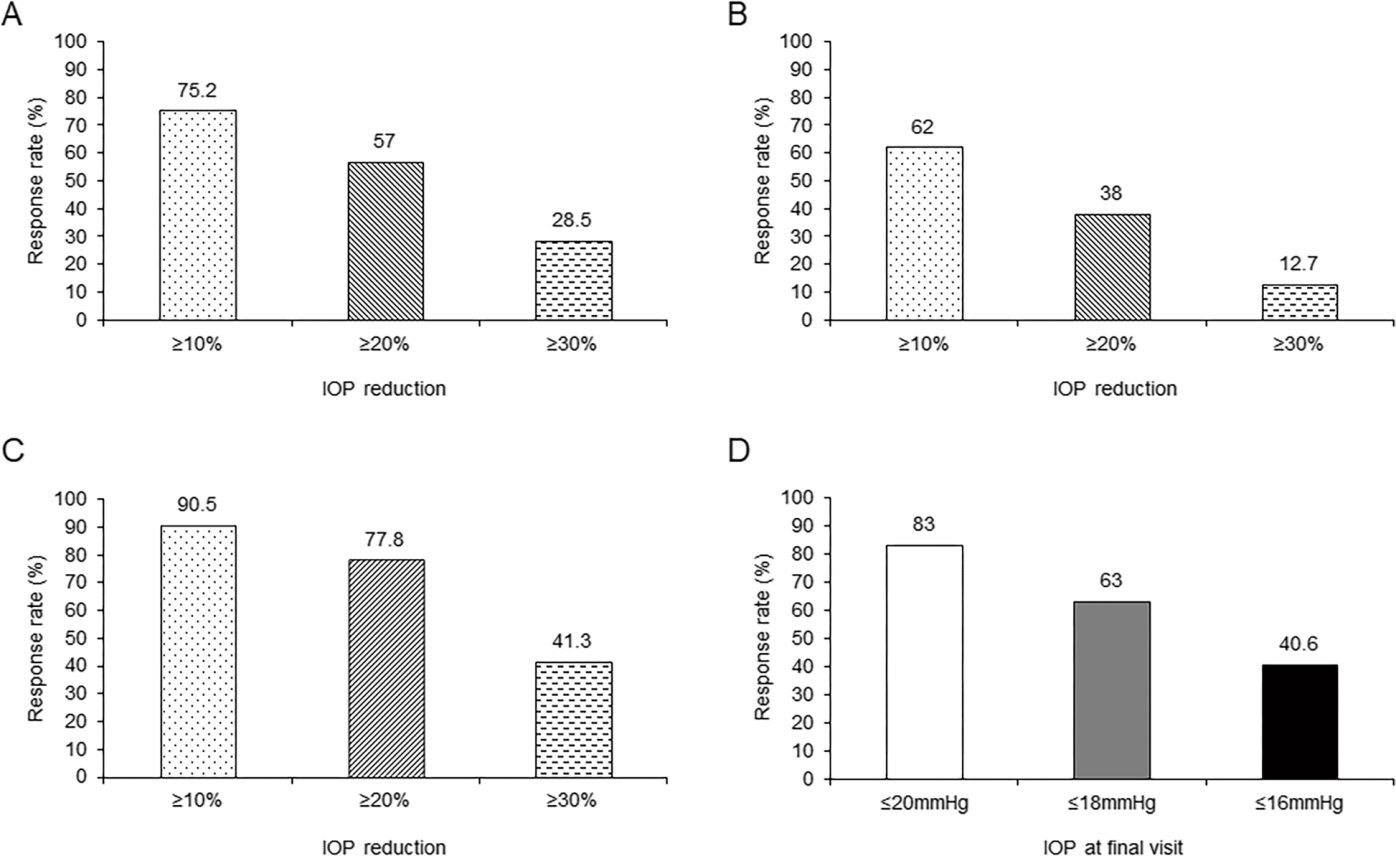
Response rate of intraocular pressure (IOP) reduction ≥ 10%, ≥ 20%, and ≥ 30% in Group A at the last visit. **A** All; **B** Baseline IOP ≤ 21 mmHg; **C** Baseline IOP ≥ 24 mmHg; **D** response rate of IOP ≤ 16 mmHg, ≤ 18 mmHg, ≤ 20 mmHg in Group A at the last visit

In group A, the Lsmeans of IOP was reduced by 5.0 mmHg (21.1%) at 1 week and 4.7 mmHg (19.8%) at 3 months of treatment. In addition, 75.2%, 57.0%, and 28.5% of the participants achieved an IOP reduction of ≥ 10%, ≥ 20%, and ≥ 30% at the final visit, respectively (Fig. 2A). In group A, the proportions of patients with an IOP reduction of ≥ 10%, ≥ 20%, and ≥ 30% at the last visit in participants with a relatively higher baseline IOP (*n* = 63, IOP ≥ 24 mmHg) were 90.5%, 77.8%, and 41.3%, respectively (Fig. 2C), and in those with a relatively lower baseline IOP (*n* = 79, IOP ≤ 21 mmHg) were 62.0%, 38.0%, and 12.7%, respectively (Fig. 2B). Furthermore, 40.6%, 63.0%, and 83.0% of the participants in group A had their IOP controlled to ≤ 16, ≤ 18, and ≤ 20 mmHg at the final visit, respectively (Fig. 2D).

**Fig. 3:**
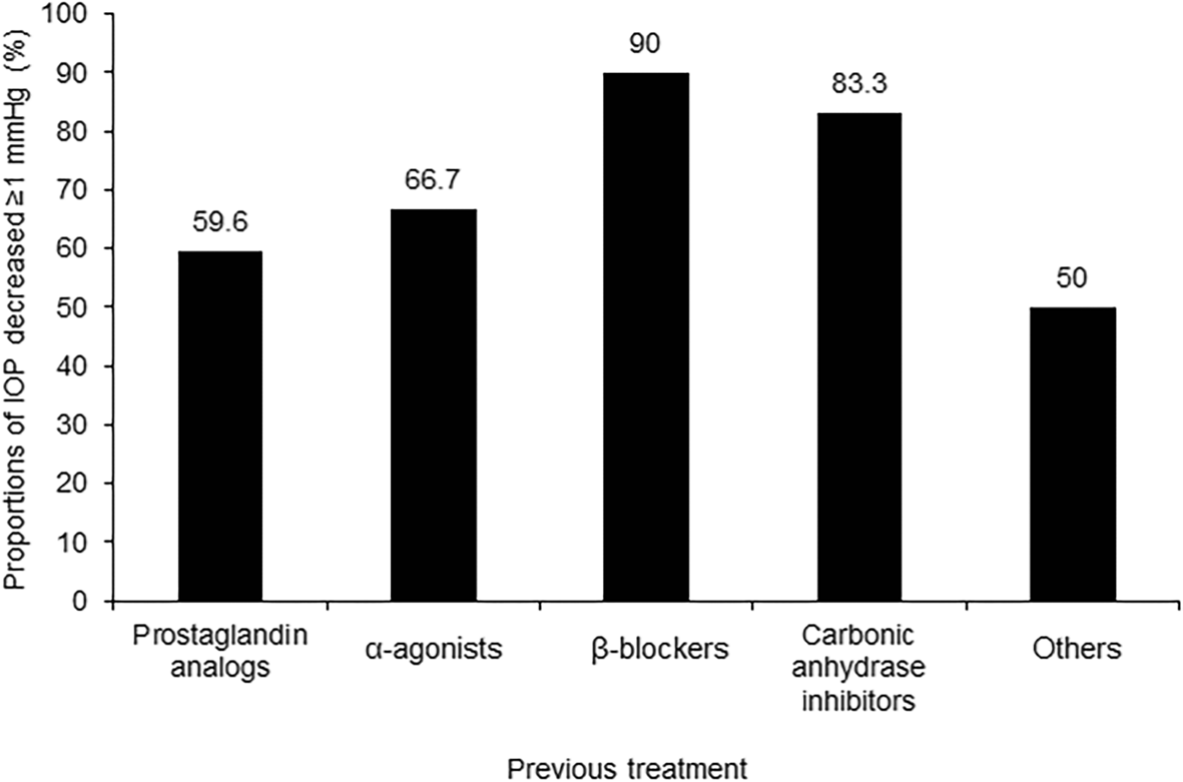
Proportions of intraocular pressure (IOP) decreased by ≥ 1 mmHg at the last visit in Groups B and C

Significant IOP reduction from baseline to 1 week, 1 month, and 3 months in group B were observed (*P* < 0.001, *P* < 0.001, and *P* = 0.006, respectively, Table 2). The Lsmeans of IOP reductions for group B was 2.8 mmHg (11.2%) at 1 week and 1.6 mmHg (6.1%) at 3 months after treatment. The proportion of participants achieving an IOP reduction of ≥ 10% at the final visit was 40.4%. Finally, 59.6% of the participants had an IOP decreased by ≥ 1 mmHg at the final visit from baseline (Fig. 3).

The Lsmeans of IOP reductions for group C were 4.1 mmHg (17.6%) at 1 week and 4.6 mmHg (20.3%) at 3 months. A significant IOP reduction from baseline was also achieved at each visit (all *P* < 0.001, Table 2). In addition, 77.4% of the participants achieved an IOP reduction of ≥ 10% at the final visit. The overall proportion of patients in group C whose IOP decreased by ≥ 1 mmHg from baseline to the final visit was 83.9%, of which the exclusive proportions for α-agonists, β-blockers, carbonic anhydrase inhibitors (CAIs), and other non-PGAs were 66.7%, 90.0%, 83.3%, and 50.0%, respectively (Fig. 3).

### Safety

In this study, 46 participants (15.7%) had 58 treatment-related AEs (TRAEs), with only one participant (0.3%) who had a systemic AE of a dry pharynx. The remaining 57 ocular TRAEs occurred in 46 participants (15.7%), of which 91.3% (42/46) were mild. There were no serious AEs related to tafluprost. The most common TRAE was conjunctival hyperemia (34/293, 11.6%), followed by ocular hyperemia (3/293, 1.0%) and ocular pruritus (3/293, 1.0%) (Table 3). Seven participants stopped using tafluprost (five in group A and two in group C) owing to ocular TRAE.

**Table 3:** Adverse events related to the study drug (SS, *N* = 293)

	Participants (%)	Events^a^
Ocular	46 (15.7)	57
Conjunctival hyperemia	34 (11.6)	37
Ocular hyperemia	3 (1.0)	3
Eye pruritus	3 (1.0)	3
Dry eye	3 (1.0)	3
Black eye	2 (0.7)	2
Eyelash growth	2 (0.7)	2
Cataract	1 (0.3)	1
Corneal abscission	1 (0.3)	1
Ocular secretion	1 (0.3)	1
Ocular sensory abnormality	1 (0.3)	1
Eye pain	1 (0.3)	1
Ocular foreign body sensation	1 (0.3)	1
Meibomian gland dysfunction	1 (0.3)	1
Systematic		
Dry throat	1 (0.3)	1

^a^ A given participant could have multiple events of the same adverse event

Tafluprost-related conjunctival hyperemia occurred in 34 patients, most of them were mild (32/34, 94.1%) and others were moderate. Four participants terminated tafluprost owing to conjunctival hyperemia, while the majority of them continued the treatment. Of the 37 conjunctival hyperemia in 34 patients, 20 (54.1%) in 19 (55.9%) patients recovered completely, and 5 (13.5%) in 5 (14.7%) patients showed improvement, while 6 (16.2%) in 6 (17.6%) patients did not improve by the end of the study.

## Discussion

This phase IV, multicenter, non-comparative, prospective study is the first post-marketing study with a relatively large sample size that evaluated tafluprost in the Chinese population. The results confirmed a satisfying IOP-lowering effect and manageable safety profile of tafluprost in Chinese patients with POAG and OH who were treatment-naïve or untreated within 1 month or with unreached target IOP after previous IOP-lowering drugs. The IOP reduction at 1 week, 1 month, and 3 months of treatment indicated a statistically significant improvement, regardless of baseline IOP.

The mean IOP of healthy Chinese was 15.0 ± 2.8 mmHg [26], lower than that of a healthy population in other races [27, 28]. Among Chinese POAG, NTG accounts for as high as 70%, with an upper limit of IOP ≤ 21 mmHg [29]. In this study, the IOP at each visit was significantly lower than baseline in all three groups. Park et al. [16] demonstrated an IOP decrement by 4.1 mmHg (24.1%) in patients with POAG and NTG after 3 months of tafluprost treatment, consistent with the results in this study. Other RCTs and observational studies also reported similar results [17, 30]. Chabi et al. reported a significant IOP decrease in patients with POAG and OH using tafluprost after 2 weeks [31]. The decrement in Lsmean after 4 weeks was 7.7–9.0 mmHg, higher than in the present study. This difference might be related to the selection of the patients, as IOP reduction is prone to be more considerable when only including patients with a baseline IOP of ≥ 24 mmHg. Nevertheless, the present study included a wider range of participants regardless of their baseline IOP level or previous treatment status to illustrate the characteristics of Chinese patients in a real-world clinical situation. The IOP reductions reported in this study were similar to previous results. A retrospective study [13] showed that in a congregation of patients with various types of glaucoma who received different treatments (naïve, switching to tafluprost, and tafluprost as add-on therapy), the average IOP decreased by 6.18 ± 4.06 mmHg after 3 months of tafluprost treatment. These results are consistent with the findings in a meta-analysis of five RCTs [32].

For the treatment-naïve patients in group A, the Lsmeans of IOP revealed a significant reduction of 5.0 mmHg (21.1%) after 1 week and was maintained at 4.7 mmHg (19.8%) after 3 months. Lanzl et al. reported an IOP reduction of 7.3 mmHg and 6.9 mmHg in patients with POAG and OH, respectively, while they had a baseline IOP of ≥ 24.4 mmHg [30]. The reduction was also observed in the phase III RCT conducted in the Chinese population, but with a larger reduction of 9.8 ± 4.0 mmHg (37.2% ± 13.4%), which was potentially caused by the lower baseline IOP in our study [25]. Nevertheless, the participants in the present study might better represent the actual situation in clinical practice. In group A, 57.0% and 28.5% of patients achieved an IOP reduction of ≥ 20% and ≥ 30% at the final visit, respectively, less than in a previous European study with a much higher baseline IOP [30]. In addition, 79 patients with a baseline IOP ≤ 21 mmHg in group A showed final-visit response rates of 62.0%, 38.0%, and 12.7% for IOP reductions of ≥ 10%, 20%, and 30%, respectively. It demonstrated that tafluprost could effectively reduce IOP regardless of baseline level and could be especially effective for most patients with NTG in clinical practice. Furthermore, 40.6%, 63.0%, and 83.0% of the patients in group A had their IOP controlled to ≤ 16, ≤ 18, and ≤ 20 mmHg after 3 months of tafluprost, while Erb et al. reported that 50.9% and 79.4% of the patients achieved ≤ 16 and ≤ 18 mmHg after much longer treatment durations [24].

For patients in group B with unreached target IOP from another PGA monotherapy, tafluprost could further reduce IOP by 2.8 mmHg (11.2%) at 1 week and 1.6 mmHg (6.1%) at 3 months. Consistent with our results, an observational study [21] also showed an average IOP decrease of 1.2 mmHg after 3 months of treatment in patients previously treated with PGAs. The minimal change can be blamed for the direct switch to tafluprost without a washout period, yet the difference is still statistically significant. In group B, 40.4% of patients achieved an IOP reduction of ≥ 10% at the final visit, similar to the response rate of 44.7% observed previously [23], suggesting the effectiveness of tafluprost in patients unresponsive to other PGAs. Elevated IOP is the most significant risk factor for optic nerve damage and visual field progression in glaucoma [33, 34]. It has been demonstrated that each 1 mmHg of IOP reduction corresponds to a 10%-19% decrease in the risk of progression in glaucomatous damage [35, 36], suggesting the importance of absolute IOP reduction during glaucoma or OH management. In the present study, 59.6% of the patients had IOP decreased by ≥ 1 mmHg at the final visit from baseline in group B, indicating that tafluprost could effectively further reduce IOP in patients previously treated with other PGAs.

The Lsmeans of IOP reductions for group C were 4.1 mmHg (17.6%) at 1 week and 4.6 mmHg (20.3%) at 3 months after treatment, much more than those reported in previous studies conducted in Japan and the Philippines [13, 23]. A similar reduction of 4.1 mmHg was also observed in a German study [37]. In addition, 77.4% of the patients in group C achieved an IOP reduction of ≥ 10% at the final visit compared with baseline. Furthermore, 66.7%, 90.0%, 83.3%, and 50.0% of the participants had their IOP decreased by ≥ 1 mmHg at the final visit after previous monotherapy of α-agonists, β-blockers, CAIs, and other non-PGAs. These results indicated that tafluprost could greatly reduce IOP despite unsatisfied outcomes from non-PGA treatment. Furthermore, a direct switch without a washout period appeared closer to the clinical practice situation. Several studies also showed IOP reductions of 3.8–5.4 mmHg when switching from non-PGAs monotherapy to tafluprost, and tafluprost provided the most IOP reduction for previous β-blockers among all prior monotherapies [24, 37], which was consistent with our data.

The total incidence of TRAE in this study was 15.7%, similar to a large Japanese prospective cohort study (18.64%) [21]. An RCT [17] reported a similar safety profile between tafluprost and latanoprost. A meta-analysis showed no statistically significant differences in safety between benzalkonium chloride-containing PGAs and preservative-free PGAs [38]. Despite the tafluprost in this study containing a relatively high concentration of benzalkonium chloride (0.1 mg/ml), neither additional TRAE nor worsened TRAE was observed. Besides, higher receptor selectivity and lower concentration of active components might also result in better safety [15]. According to previous studies, conjunctival hyperemia appeared as the most frequent TRAE in our analysis (11.6%), which is lower than other PGAs, including bimatoprost. The retrospective study in the Philippines [13] showed the most frequent TRAE conjunctival hyperemia at 15%. A phase III trial in China [25] also reported conjunctival hyperemia of 20.8%. Most of the TRAEs observed in this study were ocular-related, and only one participant displayed dry pharynx.

Two previous studies reported equivalence between latanoprost and tafluprost [17, 32], but in the present study, better efficacy was observed after switching to tafluprost. Tafluprost has a high and selective binding affinity for the prostanoid FP receptor [15]. Tafluprost can increase the mean blur rate on the optic disc [39] and improve retinal circulation [40]. Tafluprost showed an effect in relaxing rabbit ciliary artery smooth muscle, which may be due to inhibition of capacitative Ca^2+^ entry from the extracellular space [41]. This is a unique mechanism of action that differs from other PGAs. Furthermore, tafluprost can almost completely prevent ET-1-induced decrease in blood flow, and the inhibitory effect lasted longest with tafluprost. Thus, the improvement of the ocular blood flow might be superior with tafluprost than with other PGF2α analogues [42]. In addition, in the present study, tafluprost was well tolerated, and only seven patients stopped using tafluprost due to ocular TRAEs, leading to adequate dose exposure. Thus, the IOP-lowering effect is better after switching to tafluprost.

Owing to the unstandardized prior IOP-lowering regimen applied to some participants, this study was limited by the relatively insufficient sample size in the groups of treated patients, especially those receiving non-PGAs (group C). Furthermore, the 3-month follow-up was relatively short. Some patients were lost to follow-up due to the COVID-19 pandemic. Moreover, this study merely focused on patients requiring monotherapy, limiting the results in application in complex clinical settings where combined therapy and off-label use are also common circumstances. Future long-term, large-sample studies should be carried out to verify the results.

## Conclusion

Tafluprost shows stable effectiveness in lowering IOP with a manageable safety profile among Chinese patients with POAG and OH who were treatment-naïve or untreated within a month regardless of baseline IOP level or with unreached target IOP after receiving other IOP-lowering monotherapies.

## Data Availability

The datasets generated during and/or analysed during the current study are not publicly available due to we have no registered databases to store data, but are available from the corresponding author on reasonable request.

## Abbreviations

AEs: Adverse events
CIs: Confidence intervals
EMGT: Early Manifest Glaucoma Trial
FAS: Full analysis set
IOP: Intraocular pressure
LOCF: Last observation carries forward
NMPA: National Medical Products Administration
NTG: Normal-tension glaucoma
OCT: Optical coherence tomography
OH: Ocular hypertension
PGAs: Prostaglandin analogs
POAG: Primary open-angle glaucoma
RCTs: Randomized controlled trials
SS: Safety set
TRAEs: Treatment-related AEs
